# Prevalence of Bruxism in alcohol abusers: a systematic review conducted according to PRISMA guidelines and the cochrane handbook for systematic reviews of interventions

**DOI:** 10.1186/s12903-024-03862-1

**Published:** 2024-01-18

**Authors:** Vincenzo Ronsivalle, Maria Maddalena Marrapodi, Yuliia Siurkel, Marco Cicciù, Giuseppe Minervini

**Affiliations:** 1 Department of Biomedical and Surgical and Biomedical Sciences, Catania University, Catania, 95123 Italy; 2 Department of Woman, Child and General and Specialist Surgery, University of Campania, “Luigi Vanvitelli,”, Naples, 80138 Italy; 3 International European University School of Medicine, Akademika Hlushkova Ave, 42В, Kyiv, 03187 Ukraine; 4 Saveetha Dental College and Hospitals, Saveetha Institute of Medical and Technical Sciences (SIMATS), Saveetha University, Chennai, Tamil Nadu India; 5 Multidisciplinary Department of Medical-Surgical and Dental Specialties, University of Campania Luigi Vanvitelli, Naples, 80138 Italy

**Keywords:** Bruxism, Alcohol, TMD, Temporomandibular disorders, Alcohol drinking

## Abstract

**Background:**

Bruxism, a common oral parafunctional behavior characterized by the grinding or clenching of teeth, is a multifactorial condition with potentially detrimental effects on oral health and overall well-being. In recent years, there has been growing interest in understanding the relationship between bruxism and alcohol abuse, as both are prevalent issues that may share underlying factors and exacerbate each other. This systematic review, following the Preferred Reporting Items for Systematic Reviews and Meta-Analyses (PRISMA) statement, aims to evaluate the frequency of bruxism among individuals with alcohol abuse.

**Methods:**

A comprehensive search of electronic databases, including PubMed, Lilacs, Scopus and Web of Science, will be conducted to identify relevant studies published up to the knowledge cutoff date in January 2023. The search strategy will include keywords related to bruxism, alcohol abuse, and their synonyms. Inclusion criteria will encompass original research studies, such as observational, cross-sectional, cohort, and case-control studies, as well as clinical trials, that examine the relationship between bruxism and alcohol abuse. Two independent reviewers will perform the study selection, data extraction, and quality assessment, with discrepancies resolved by consensus.

**Results:**

The systematic review will present a summary of the identified studies, including the study design, characteristics of the study populations, and key findings related to the association between bruxism and alcohol abuse. The potential mechanisms underlying this relationship will also be explored. Subgroup analyses and the quality of evidence will be assessed. Finally, the implications of this association for clinical practice and further research will be discussed.

**Conclusions:**

This systematic review will contribute to a better understanding of the interplay between bruxism and alcohol abuse, shedding light on potential risk factors, mechanisms, and clinical implications. The findings may have significant implications for the prevention, management, and treatment of bruxism, particularly in individuals with a history of alcohol abuse.

## Introduction

Bruxism, a condition characterized by the grinding or clenching of teeth, has long been a subject of scientific inquiry and clinical concern [1]. It is a multifaceted dental and neurological disorder that can lead to a variety of oral and systemic health issues [2]. Concurrently, alcohol abuse represents a significant public health challenge worldwide, with numerous detrimental effects on physical and mental well-being [3]. The convergence of bruxism and alcohol abuse in an individual’s life introduces a complex interplay of factors that can exacerbate the consequences of both conditions, leading to a range of serious health concerns [4–8]. This introduction aims to provide a comprehensive overview of bruxism, alcohol abuse, and the intricate relationship between the two, shedding light on the underlying mechanisms, clinical implications, and the need for further research in this area.

Bruxism, derived from the Greek word “brychein,” meaning “to grind or gnash the teeth,” is a repetitive jaw-muscle activity involving clenching or grinding of the teeth [9]. It is a condition that can manifest during wakefulness (awake bruxism) and during sleep (sleep bruxism) [9]. Awake bruxism is often characterized by teeth clenching and grinding during periods of wakefulness, which may be subconsciously or consciously driven [9]. Sleep bruxism, on the other hand, occurs during various sleep stages and is primarily characterized by rhythmic or non-rhythmic contractions of the masticatory muscles and grinding of the teeth [9].

Bruxism is a relatively common condition, with a prevalence that varies across populations. Prevalence rates reported in the literature range from 5 to 20% for awake bruxism and 8–13% for sleep bruxism [10]. Although it can affect individuals of all ages, bruxism is most frequently observed in children, adolescents, and adults [10]. The exact reasons for the variations in prevalence remain unclear but are likely influenced by genetic, psychosocial, and environmental factors [1].

The exact etiology of bruxism is not fully understood, and it is considered a multifactorial condition with a combination of genetic, psychological, and environmental contributors [9]. While awake bruxism is often associated with stress, anxiety, and emotional tension, sleep bruxism is thought to result from a complex interplay between genetic predisposition, central nervous system dysfunction, and peripheral factors [11]. Neurological mechanisms involving alterations in the brainstem, particularly the mesencephalic trigeminal nucleus, have been proposed as a potential trigger for sleep bruxism [12].

Bruxism can lead to various clinical manifestations and health consequences. Patients with bruxism often report symptoms such as jaw pain, muscle fatigue, headaches, tooth sensitivity, and worn-down teeth [13]. Over time, the mechanical forces associated with bruxism can result in dental complications, including enamel erosion, chipped or fractured teeth, and temporomandibular joint disorders [14, 15]. Furthermore, the continuous grinding and clenching of teeth can also have systemic effects, potentially contributing to sleep disturbances, chronic pain, and decreased overall quality of life [9].

Alcohol abuse, characterized by the excessive and harmful consumption of alcoholic beverages, is a significant global public health concern [16]. The harmful use of alcohol has far-reaching consequences on both individual and societal levels, affecting physical, mental, and social well-being [16, 17]. Understanding the prevalence, etiology, and consequences of alcohol abuse is essential in comprehending its intersection with bruxism.

Alcohol abuse is a pervasive issue that affects millions of individuals worldwide. Its prevalence varies across countries and demographics, with factors such as cultural norms, social environment, and socioeconomic status playing significant roles [18]. Men are generally more likely to engage in alcohol abuse than women, and the prevalence tends to increase with age, particularly among young adults [19].

The etiology of alcohol abuse is multifaceted and complex, involving genetic, psychological, social, and environmental factors [20]. Genetic predisposition, family history of alcohol use disorders, and neurological factors have been implicated in the development of alcohol abuse [20]. Psychological factors, such as stress, anxiety, and depression, often contribute to alcohol misuse as individuals may turn to alcohol as a coping mechanism. Social factors, including peer pressure and societal acceptance of alcohol consumption, also influence alcohol abuse [20].

The clinical manifestations of alcohol abuse are diverse and can range from acute intoxication to chronic health conditions [21]. Short-term consequences may include impaired judgment, motor coordination, and memory, as well as an increased risk of accidents and injuries [21]. In the long term, alcohol abuse can lead to chronic health issues such as liver disease [22, 23], cardiovascular disease [24], neurocognitive deficits [16, 25, 26], and mental health disorders [20]. Furthermore, alcohol abuse can have detrimental social consequences, affecting relationships, employment, and overall quality of life [9].

Comprehending the intricate relationship between bruxism and alcohol abuse is of paramount importance for professionals in dentistry, psychology, and addiction medicine. The concurrent presence of these conditions can trigger complex mechanisms that intensify the clinical symptoms and outcomes associated with both.

One key aspect of the intersection between bruxism and alcohol abuse is the presence of shared risk factors. Stress, anxiety, and psychological distress are recognized contributors to both conditions [4]. Individuals experiencing high levels of stress may resort to alcohol as a coping mechanism, which, in turn, can exacerbate their risk of developing alcohol abuse [20]. Simultaneously, stress and emotional tension are well-established triggers for awake bruxism, highlighting the potential for common precipitating factors [20].

Sleep bruxism is particularly intriguing in the context of alcohol abuse. While the etiology of sleep bruxism is multifactorial, alcohol consumption has been suggested as a potential trigger. The depressant effects of alcohol on the central nervous system may alter sleep architecture and disrupt the normal regulation of motor activity during sleep [21]. This disruption could lead to increased occurrences of sleep bruxism events, potentially causing more severe dental and musculoskeletal consequences in individuals who abuse alcohol [4].

The convergence of bruxism and alcohol abuse introduces the potential for compounding health consequences. For example, the continuous grinding and clenching of teeth in individuals with bruxism can be further intensified by the motor disturbances associated with alcohol intoxication during sleep [9]. This intensified bruxism may lead to more severe dental damage, such as fractured or eroded teeth, increasing the financial and health burdens on affected individuals.

Both bruxism and alcohol abuse can significantly affect an individual’s quality of life, and their co-occurrence may lead to a disproportionate decrease in well-being [9]. The combined impact of dental complications, chronic pain, and sleep disturbances can result in poor sleep quality, reduced daily functioning, and emotional distress [9]. Furthermore, the detrimental social consequences of alcohol abuse may be amplified when combined with the visible signs of bruxism, potentially leading to social stigmatization and isolation [4].

The intersection of bruxism and alcohol abuse presents a compelling area of research and clinical concern.

This systematic review aimed to assess the prevalence of Bruxism in Alcohol abusers.

## Materials and methods

### Eligibility criteria

All documents were assessed for eligibility based on the Population, Exposure, Comparator, and Outcomes (PECO) model [27].

P) Participants consisted of human subjects.
E) The Exposure consisted of exposition to Alcohol abuse.
C) The Comparison was with no-Alcohol abusers.
O) The Outcome consisted of Prevalence of Bruxism in alcohol abusers.

Only research papers that presented conclusive data upon completion of the intervention were considered for inclusion. The exclusion criteria encompassed: (1) patients diagnosed with rheumatic diseases or chronic inflammatory disorders (e.g., rheumatoid arthritis, juvenile, idiopathic arthritis, psoriatic arthritis); (2) patients experiencing dental pain; (3) patients with psychiatric disorders; (4) individuals having a history of facial conditions; (5) studies involving patients with partial dentures; (6) cross-over study designs; (7) studies not documented in English; (8) unavailability of full-text materials (e.g., posters, conference abstracts); (9) studies involving animal subjects; (10) review articles (topical or systematic); 11) case reports or series.

### Search strategy

We systematically searched Web of Science, PubMed, Scopus and Lilacs for articles published from the inception until November 20, 2023. We followed the strategy following the strategy reported in Table 1. Furthermore, we manually searched the references and previous systematic reviews on a similar topic.

**Table 1:** Search strategy

***PubMed***
Search: **((alcohol) AND (bruxism)) NOT (review)** Filters: **English**
(((“alcohol s”[All Fields] OR “alcoholate”[All Fields] OR “alcoholates”[All Fields] OR “alcohols”[MeSH Terms] OR “alcohols”[All Fields] OR “ethanol”[MeSH Terms] OR “ethanol”[All Fields] OR “alcohol”[All Fields]) AND (“bruxism”[MeSH Terms] OR “bruxism”[All Fields])) NOT (“review”[Publication Type] OR “review literature as topic”[MeSH Terms] OR “review”[All Fields])) AND (english[Filter])
***Scopus***
TITLE-ABS-KEY (bruxism AND alcohol)
***Web of Science***
ALL=(bruxism)) AND ALL=(alcohol)
***Lilacs***
alcohol [Palavras] and BRUXISM [Palavras]

This systematic review was conducted according to the guidance of the Cochrane Handbook for Systematic Reviews of Interventions and the Preferred Reporting Items for Systematic Reviews (PRISMA) guidelines 2020. The systematic review protocol has been registered on the International Prospective Register of Systematic Reviews (PROSPERO) with the provisional PROSPERO number 483272.

### Data extraction

Two reviewers (VR and GM) extracted the data from the included studies using customized data extraction on a Microsoft Excel sheet. In case of disagreement, a consensus was reached through a third reviewer (MC). The following data were extracted: (1) First author; (2) Year of publication; (3) Nationality; (4) Number of study participants (5) Diagnostic tool; (6) Clinical relevance.

### Quality assessment

Two reviewers (GM and VR) assessed the risk of bias using Robins-E tool. Any disagreement was discussed until a consensus was reached with a third reviewer (MC).

### Quality assessment

For this review, the Risk of Bias in Non-randomized Studies of Interventions (ROBINS-E) tool was utilized to assess the risk of bias in the included studies. The ROBINS-E tool provides a structured framework to evaluate potential biases in non-randomized studies. The assessment process involved independent evaluation by two or more reviewers for each study included in the systematic review. The reviewers were trained in using the ROBINS-E tool and followed the guidelines to evaluate the risk of bias across seven domains: confounding, selection of participants, classification of interventions, deviations from intended interventions, missing data, measurement of outcomes, and selection of reported results. To enhance the objectivity and consistency of the assessments, any discrepancies or disagreements between reviewers were resolved through discussion and consensus. In cases where consensus could not be reached, a third reviewer was involved to make the final determination. The bias assessment using ROBINS-E provided a comprehensive evaluation of the potential biases in the included non-randomized studies. It helps to identify the strengths and limitations of the evidence base, contributing to the overall assessment of the quality and reliability of the findings. By considering the risk of bias, the review authors can make more informed interpretations and draw conclusions based on the available evidence.

## Results

### Study characteristics

**Fig. 1:**
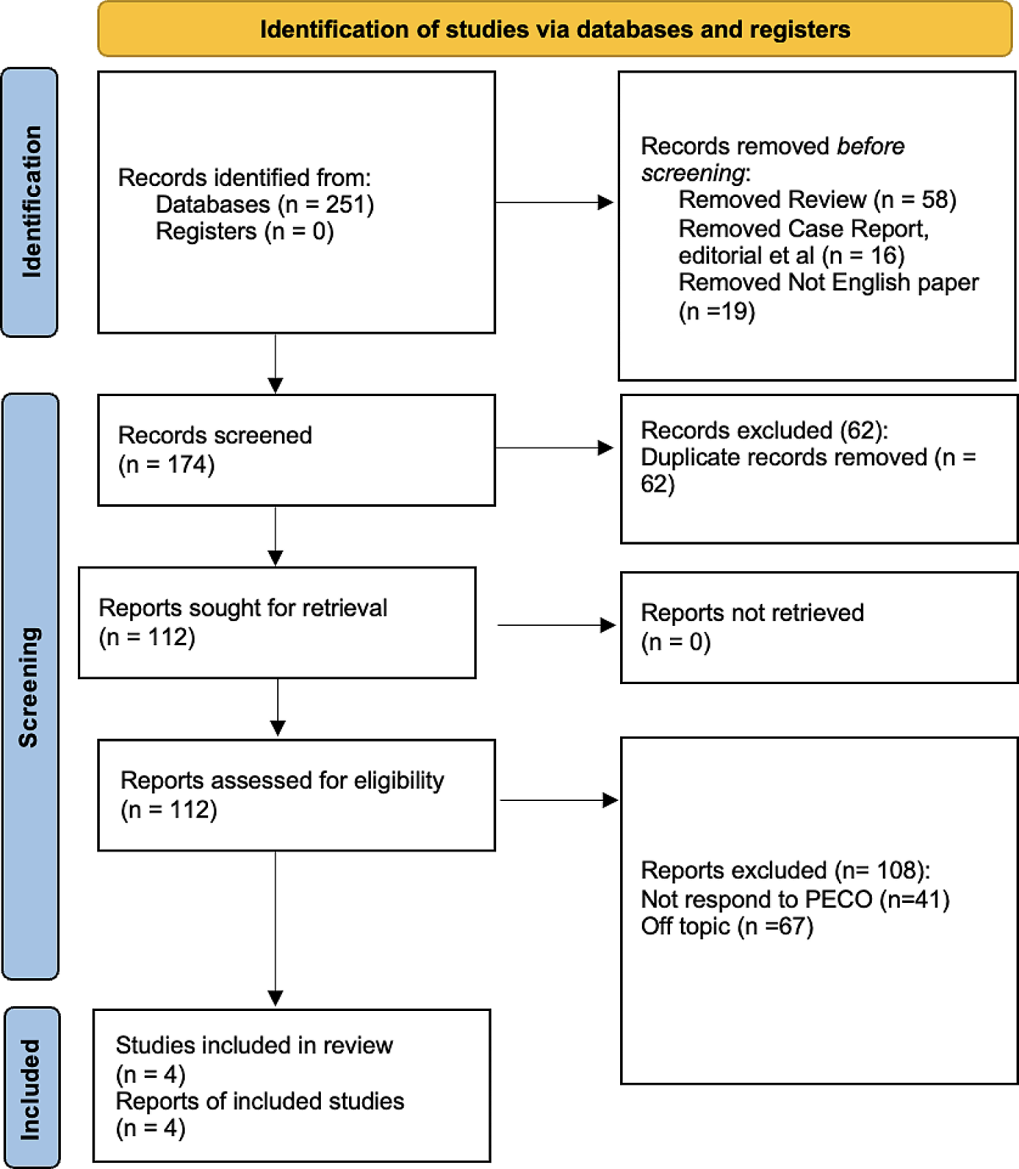
Prisma Flowchart

Two hundred fifty-one studies were identified at the end of the research. As illustrated by the PRISMA 2020 flowchart in Fig. 1, we chose only four studies to draw up the present systematic survey. We excluded 93 articles before the screening: 58 were reviews, 16 were case reports and editorials, and 19 were not in English. The remaining articles (*n* = 174) were selected for the title and abstract screening to evaluate whether they meet the PECO criteria. Sixty-two articles were excluded as duplicates and one hundred-twelve records were assessed for eligibility. Among these, 108 were excluded: 41 did not respond to PECO, and 67 were off-topic. The included studies have been published between 1979 and 2022. A total of 4 studies were included in the review. All studies compare the prevalence of bruxism among human subject who abuse alcohol in comparison to those who are moderate consumers of alcohol or abstain from its consumption. Table 2 summarizes the main characteristics of all the studies included in the present systematic review (as reported in the paragraph data extraction).

**Table 2:** Syntesis of the studies included in this systematic review

Author	Year	Nationality	Study type	Number of subjects	Clinical relevance
Rintakoski et al.	2013	Finland	Cohort study	12 502 subjects	Increasing alcohol intake raised the risk for weekly bruxism
Frosztega et al.	2022	Poland	Prospective-observattional study	133 subjects	Alcohol consumption has no significant influence on bruxism parameters.
HArtmann	1979	USA	Observational study	4 subjects	Alcohol consumption is related to nocturnal bruxism episodes
Miettinen	2017	Finland	Epidemiologic-cross-sectional study	8 678 subjects	Increased frequency of alcohol consumption is associated wit TMD symptoms including bruxism

### Main findings

The included subjects in this review were 21.317. Among them, 12,639 subject were included in studies evaluating the influence of alcohol and smoking inpatient with bruxism, while the other 8,678 subject were included in a study evaluating the influence of alcohol and smoking in patients with TMD. The average age of all included subject was 36 years, but only Hartmann’s study did not provide any data regarding the age of the subjects included in the study, indicating them only as adult patients.

The number and demographic characteristics of the enrolled patients in the four studies are presented in Table 2.

Rintakoski et al. [28] study investigate the possible independent effects of drinking alcohol and coffee consumption on the occurrence of bruxism. A total of 12,502 twin individual patients, born between 1930 and 1957, responded to a questionnaire sent in 1990 as part of the third survey of the longitudinal Finnish Twin Cohort study. The mean age of respondents in 1990 was 44 years and the response rate was 77%. Information about bruxism was available for 10,229 individuals of which: 4% reported bruxism weekly, 4% monthly, 19% rarely and the rest 72% never. Coffee consumption was common, with 10% reporting consumption of more than eight cups daily. Among those with bruxism, heavy drinking was more prevalent in men (19.5%) than in women (10.3%). Abstainers were more common among women (18.9%), and the mean alcohol drinks per day were 1.3 for men and 0.46 for women. Smoking was less common among abstainers, weekly bruxism was less frequent among abstainers and individuals with moderate coffee consumption (< 8 cups per day) compared to heavy drinkers and those consuming more than 8 cups per day, respectively. Multinomial logistic regression models showed associations between coffee consumption, heavy drinking, binge drinking, passing-out due to alcohol, and weekly bruxism, even when adjusted for smoking status and other factors. Pairwise analyses within twin pairs supported the independent effects of coffee and alcohol on bruxism, suggesting that shared genes or environments did not significantly confound the results.

Frosztega et al. [29]study evaluate sleep bruxism intensity in tobacco smokers and alcohol drinkers. The aim of the study was to investigate the relationship between bruxism intensity in tobacco smokers and alcohol drinkers using polysomnographic assessments. A total of 133 adults with mean age of 47.89 ± 16.57 years, underwent full-night audio- and video-polysomnography. The study group was divided into smoker and nonsmoker groups as well as drinker and non-drinker groups. Those who consumed both alcohol and tobacco contributed to 14% of the study group. The following comorbidities were observed among the participants: myocardial infarction (*n* = 7), stroke history (*n* = 6), hypertension (*n* = 52), diabetes (*n* = 24), and ischemic heart disease (*n* = 10). The results of the polysomnographic analysis confirmed that tobacco smoking has a significant effect on SB. Tobacco smokers showed increased bruxism intensity (5.50 ± 4.71 vs. 3.83 ± 3.26, *p* < 0.05), especially the mixed phenotype (0.93± 1.00 vs. 0.59 ± 0.59, *p* < 0.05), in the N1 sleep stage (22.84 ± 20.45 vs. 15.66 ± 13.60, *p* < 0.05) and the non-supine position (4.93 ± 5.56 vs. 2.50 ± 2.31, *p* < 0.05). They also showed a higher number of bruxism episodes with arousal compared with nonsmokers (2.91 ± 2.83 vs. 1.61 ± 1.49, *p* < 0.05), indicating increased sleep fragmentation. However, no significant effect of alcohol on SB intensity was observed, and the bruxism episode index was similar in alcohol drinkers and nondrinkers. In addition, electrolyte disturbances and lipid disorders were evaluated. Compared with nonsmokers, tobacco smokers showed a higher concentration of plasma triglycerides (177.67 ± 106.9 vs. 129.18 ± 65.61) and lower levels of iron and magnesium (96.68 ± 43.58 vs. 123.83 ± 52.36 and 1.85 ± 0.22 vs. 1.96 ± 0.21, respectively), however, no significant differences were observed between alcohol drinkers and nondrinkers [30–34].

In his study, Hartmann [35] followed four adult patients, all moderate “social” drinkers, for periods ranging from four to 12 months. Each patient exhibited a history of moderate bruxism over several years. The study found a close correlation between episodes of bruxism, reported by bed partners, and alcohol intake. On days without alcohol consumption, bruxism was reported as absent or mild, while days with moderate or heavy alcohol intake resulted in audible bruxism disturbing bed partners. One patient’s case demonstrated a consistent parallel between bruxism and alcohol usage over alternating one-week periods of abstinence and moderate consumption.

Miettinen et al. study evaluate the prevalence of TMD symptoms and their associations with alcohol and smoking habits in young Finnish adults. The total sample consisted of 8,678 conscripts (8,530 men and 148 women, response rate 62.8%) born between 1990 and 1992 (mean age 19.6 years). Data on TMD symptoms, health behavior, and background/demographic factors were acquired by using a questionnaire. Self-reported facial pain and symptoms of TMD were used as outcome variables. The frequency of smoking and consumption of alcohol and snuff were used as explanatory variables. This study investigated the prevalence of temporomandibular disorder (TMD) symptoms, highlighting TMJ clicking and jaw pain as the most common. Among males, the self-reported TMD symptoms ranged from 5.8% (difficulty in jaw opening) to 27.8% (TMJ clicking). Females generally exhibited higher prevalence rates for various TMD symptoms, with facial pain at 13.6% for males and 14.9% for females. Jaw pain prevalence was 25.3% for males and 33.8% for females. Female conscripts had a higher prevalence of TMD symptoms, except for TMJ clicking. Statistically significant gender differences were observed for TMJ pain at jaw rest and difficulty in jaw opening. TMD symptoms were significantly more prevalent among those reporting toothache, smokers, and individuals with higher alcohol consumption. Snuff users also showed higher prevalence rates for most TMD symptoms. Logistic regression analysis revealed that heavy smoking over 20 cigarettes/day), frequent alcohol use, and snuff consumption significantly increased the risk of various TMD symptoms, while toothache exhibited the strongest association.

### Quality assessment and risk of bias

**Fig. 2:**
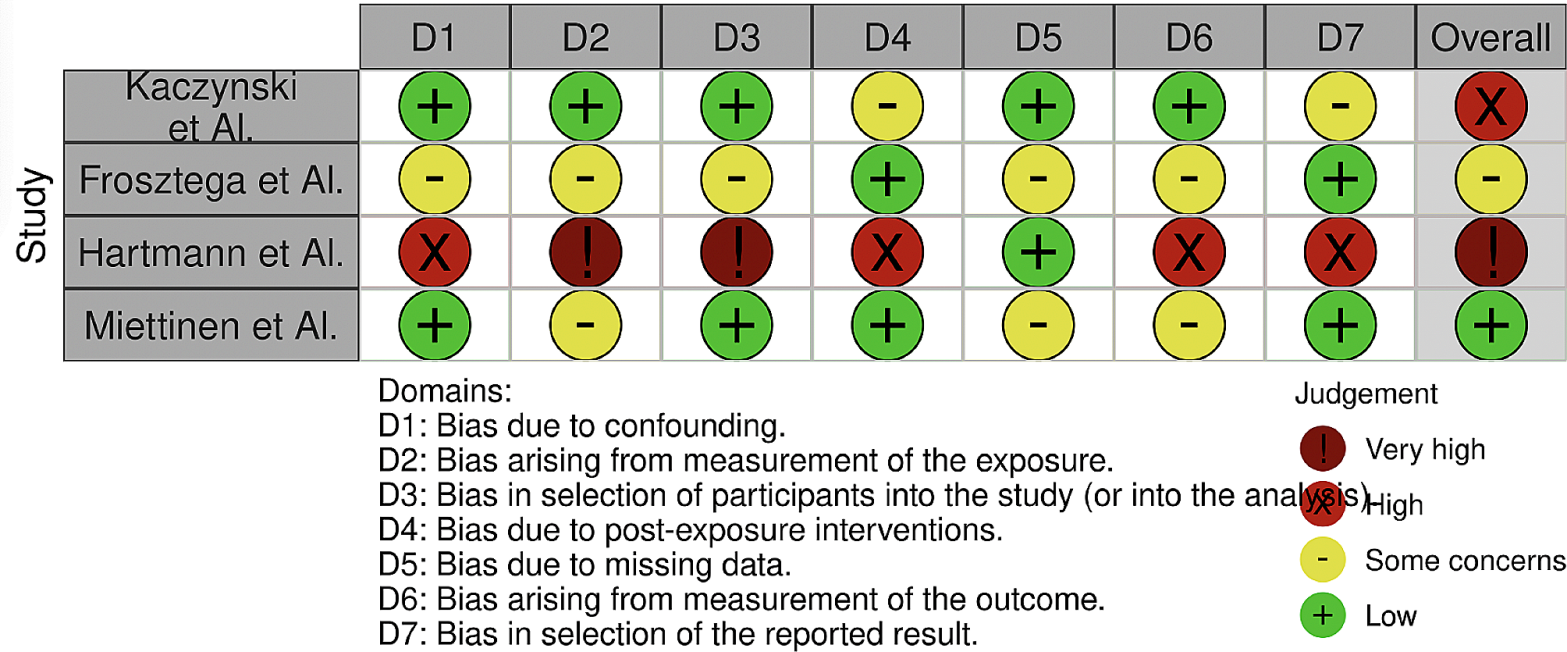
Risk of bias domain

The bias was assessed with the Robins-E tool and reporting in the Fig. 2. With regard to the bias due to confounding only the study by Kaczynski shows a low risk of bias. The bias due to measurement bias is low in almost all studies. 2 studies show a low risk of bias due to participant selection.

Also 1 study show a low bias due to measurements, although they were carried out by means of questionnaires. The bias due to the presentation of results is negligible and low. We can say as a final judgement that the overall bias of the four studies is for one low, high for one, very high for one and with some concerns for the Frosztega.

## Discussion

Results from four studies was demonstrating various associations between alcohol, smoking, and bruxism or TMD; they are resumed in Table 2. Bruxism and alcohol abuse are two distinct but interrelated issues that have garnered attention within the medical and dental research communities [35]. Bruxism, the repetitive and involuntary grinding or clenching of teeth, is a common oral parafunctional habit with potentially serious consequences for oral and overall health [28]. Alcohol abuse, on the other hand, is a widespread public health concern associated with a range of physical and psychological consequences [28]. In fact, This can lead to severe health consequences, including liver damage, impaired cognitive function, and increased risk of mental health disorders such as depression and anxiety. Additionally, it contributes to social problems, escalates the risk of accidents and injuries, and poses economic burdens on individuals and society as a whole [36]. This discussion aims to explore the potential connections between bruxism and alcohol abuse from a scientific perspective, highlighting the relevant research findings and underlying mechanisms.

Prevalence and Co-Occurrence: Bruxism and alcohol abuse both represent significant health issues. Bruxism occurs in varying degrees among the general population, with an estimated prevalence of approximately 8–31%. It can manifest as either awake bruxism or sleep bruxism. Alcohol abuse, characterized by excessive and harmful patterns of alcohol consumption, affects a substantial portion of the global population. Studies suggest that these two conditions may co-occur more frequently than expected by chance, raising questions about their potential relationship [28, 29, 35, 37].

Several neurobiological mechanisms are thought to underlie the connection between bruxism and alcohol abuse [29]. One key factor is the modulation of the central nervous system (CNS) by alcohol. Alcohol exerts a depressant effect on the CNS, affecting neurotransmitter systems, such as gamma-aminobutyric acid (GABA) and glutamate, which play a role in regulating muscle activity and sleep patterns. This CNS modulation may contribute to the increased likelihood of bruxism episodes during and after alcohol consumption [29]. Stress is a significant risk factor for both bruxism and alcohol abuse [4]. Individuals often turn to alcohol as a means of coping with stress and anxiety. However, alcohol can impair the body’s ability to respond to stress effectively, potentially exacerbating anxiety and causing or intensifying bruxism episodes [4, 38–41]. The cyclical relationship between stress, alcohol use, and bruxism further complicates the picture.

Sleep disturbances are common in individuals with alcohol abuse disorders and those who experience bruxism. Alcohol disrupts normal sleep patterns, leading to poor sleep quality, including disturbances in rapid eye movement (REM) sleep [29]. These sleep disturbances can potentially trigger or exacerbate sleep bruxism episodes. Additionally, individuals who engage in alcohol abuse may be more prone to nocturnal bruxism due to the impact of alcohol on muscle relaxation and CNS function [14].

Bruxism, when left unmanaged, can result in dental issues such as tooth wear, fractures, and temporomandibular joint disorders [15, 42–45]. The association between alcohol abuse and bruxism raises concerns about compounded dental and health consequences in individuals who experience both conditions. Proper management of bruxism is crucial to prevent the progression of dental problems and mitigate the impact of alcohol-induced bruxism episodes [28, 35].

Understanding the potential interplay between bruxism and alcohol abuse is vital for healthcare professionals. Dental practitioners, in particular, should be aware of the potential risk factors, symptoms, and consequences associated with both conditions. Addressing bruxism in individuals with a history of alcohol abuse may require a multidisciplinary approach that encompasses both dental and behavioral interventions.

The relationship between bruxism and alcohol abuse is complex and multifaceted. While research on this topic is ongoing, it is evident that both conditions have overlapping risk factors and mechanisms that warrant further investigation. Recognizing the potential connection between bruxism and alcohol abuse can lead to more effective prevention and treatment strategies for individuals at risk, ultimately promoting better oral and overall health outcomes.

Behavioral interventions play a crucial role in addressing both bruxism and alcohol abuse. Cognitive-behavioral therapy (CBT) has been employed to manage alcohol abuse by helping individuals recognize and modify the triggers and behaviors associated with excessive drinking [46]. Similarly, CBT techniques can be adapted to address bruxism by identifying and modifying the underlying stressors and behaviors that contribute to teeth grinding [47]. These interventions may also address the maladaptive coping mechanisms that link the two conditions.

Pharmacological interventions may be considered in the management of bruxism and alcohol abuse. For bruxism, muscle relaxants, such as benzodiazepines, have been prescribed in some cases to reduce muscle tension [48, 49]. However, these medications must be used cautiously due to their potential for abuse, particularly in individuals with a history of alcohol abuse. Medications to assist in alcohol addiction treatment, such as disulfiram or naltrexone, can be part of a comprehensive approach to managing alcohol abuse [50–53].

Regular monitoring and screening for both conditions are essential, especially in individuals who exhibit risk factors or symptoms. Dental professionals should be vigilant in identifying signs of bruxism, such as tooth wear and facial muscle discomfort. Primary care providers and addiction specialists must routinely screen for alcohol abuse, recognizing that it may be intertwined with bruxism [37].

Promoting holistic health and well-being is integral to addressing the interconnected issues of bruxism and alcohol abuse. Encouraging a healthy lifestyle that includes stress reduction techniques, regular exercise, and balanced nutrition can help mitigate the risk factors associated with both conditions. Implementing these practices can enhance an individual’s overall physical and psychological well-being [9].

The link between bruxism and alcohol abuse is an evolving area of research, and further investigation is required to better understand the complex interplay between these two conditions. Longitudinal studies and controlled experiments can provide deeper insights into causality, prevalence, and the most effective treatment approaches. Identifying specific genetic, neurobiological, and environmental factors that contribute to this relationship is also an important avenue for future research.

The review, while extensive, faces limitations. These include reliance on a limited number of diverse studies, potential biases within these studies, language constraints affecting study inclusion, and limited generalizability due to specific study populations. An overcoming these limitations in future studies is crucial for a more comprehensive understanding of their relationship.

## Conclusion

Alcohol abuse independently impacts the occurrence of nocturnal bruxism episodes. Understanding the relationship between bruxism and alcohol abuse emphasizes the importance of a unified approach for effective management. Recognizing this interplay allows for better prevention and treatment strategies, benefiting oral and overall health. Healthcare professionals can employ a comprehensive, patient-centered framework to address these intertwined conditions more effectively. Overall, the future prospectives involves a multidisciplinary approach, personalized interventions, increased awareness, and continued research efforts to effectively manage the interplay between bruxism and alcohol abuse for the betterment of individuals’ oral and overall health.

## Data Availability

All data generated or analysed during this study are included in this published article.
